# Synthesis, structure and physical properties of the new layered oxyselenides Bi_2_LnO_4_Cu_2_Se_2_ (Ln = rare earth)

**DOI:** 10.1098/rsos.201078

**Published:** 2020-10-28

**Authors:** Shugang Tan, Chenhao Gao, Cao Wang, Qiang Jing, Tong Zhou, Guangchao Yin, Meiling Sun, Fei Xing, Rui Cao, Yuping Sun

**Affiliations:** 1 School of Physics and Optoelectronic Engineering, Shandong University of Technology, Zibo 255000, People's Republic of China; 2 Office of International Cooperation and Exchange, Shandong University of Technology, Zibo 255000, People's Republic of China

**Keywords:** layered oxyselenides, solid-state reaction, band structure

## Abstract

We have synthesized a new series of layered oxyselenides Bi_2_LnO_4_Cu_2_Se_2_ (Ln=Nd, Sm, Eu, Dy, Er, Yb). Their crystal structures and physical properties were studied through X-ray diffraction, electric transport measurements, bulk magnetization and first-principle calculation. All these compounds have a tetragonal structure with space group I4/mmm. They exhibit hole-type metallic behaviours which is also verified by the DFT calculation. The new Bi_2_LnO_4_-type block in these compounds may give people some enlightenment in synthesizing new iron-based superconductors or other layered compounds.

## 1. Introduction

Since the discovery of superconductivity, people have been trying to find new superconductors with application values. Compounds with layered structure have always attracted people's attention because of their rich properties. Especially in recent years, many layered structural compounds have been found to exhibit superconductivity, such as cuprates superconductors [1], iron-based superconductors [2] and BiS_2_-based superconductors [3]. It has become one of the most important ways to search for new superconductors by exploring novel layered structural compounds.

Oxychalcogenides tend to adopt layered structure, due to the different sizes and coordination requirements of the oxide and the heavier chalcogenide anions [4]. Oxychalcogenides have been extensively studied because of their novel electronic or magnetic properties and intriguing structure features. Generally, TMCh-based (TM = Cu, Ag; Ch = S, Se, Te) oxychalcogenides dominated the known chemistry of oxychalcogenides, which possess very diverse and interesting structures and exhibit useful physical and chemical properties [4–9]. LnOTMCh (Ln = lanthanide) is a typical class of layered oxychalcogenides. The formulation as LaOCuS emphasizes the occurrence of two fairly distinct layer types, formally [LaO]^+^ and [CuS]^+^. The analogue BiCuSeO has attracted much attention in the thermoelectric field [10–13]. Another important type is represented by the Sr_2_MO_2_Cu_2_Ch_2_, the structure of which was first described for the oxide antimonide Sr_2_Mn_3_Sb_2_O_2_ [14–20]. These layered types have great flexibility and are each merely the most common members of the structural homologous series. In 2008, the discovery of Fe-based superconductor opens a new chapter in the research of high-temperature superconductivity. The common feature of iron-based superconductors is that they have Fe_2_Pn_2_ antifluorite layer. Many of the compounds with CuSe layers have analogues with FeAs layers, and there could be iron arsenides with this structure. In 2002, Evans *et al.* reported a new family of layered oxyselenide Bi_2_LnO_4_Cu_2_Se_2_, and five compounds were synthesized (Ln = Y, Gd, Sm, Nd, La) [21]. However, the physical properties of this series of oxyselenide are still unstudied. Recently, we studied the physical and electrical properties of Bi_2_YO_4_Cu_2_Se_2_, which exhibits quasi-two-dimensional metallic behaviour [22]. More compounds are needed to enrich this class of materials.

In this work, we report the synthesis, structure and physical properties several new compounds of the series Bi_2_LnO_4_Cu_2_Se_2_ (Ln = Sm, Nd, Eu, Dy, Er, Yb), which could give people some enlightenment in synthesizing new iron-based superconductors or other layered compounds.

## 2. Experimental details

Bi_2_LnO_4_Cu_2_Se_2_ sample was prepared by reacting a stoichiometric mixture of Bi_2_O_3_, Ln_2_O_3_, Bi, Cu and Se. The chemical equation can be written as

$$5\text{B}{\text{i}}_{2}{\text{O}}_{3}+3\text{L}{\text{n}}_{2}{\text{O}}_{3}+2\text{Bi}+12\text{Cu}+12\text{Se}\to 6\text{B}{\text{i}}_{2}\text{Ln}{\text{O}}_{4}\text{C}{\text{u}}_{2}\text{S}{\text{e}}_{2}.$$

The raw materials were mixed and ground thoroughly in an agate pestle and mortar, and then the mixture was pressed into pellets under 12 MPa. The pellets were placed into dried alumina crucibles and sealed under vacuum (less than 10^−4^ Pa) in the silica tubes which had been baked in a dry box for 1–2 h at 150°C. The ampoules were heated to 830°C with 1°C min^−1^ and maintained at this temperature for 24 h. Finally, the furnace was shut down and cooled to room temperature naturally. The obtained samples were reground, pelletized and heated for another 24 h at 830°C followed by furnace cooling. The X-ray powder diffraction patterns were recorded at room temperature on a Panalytical diffractometer (X'Pert PRO MRD) with Cu K*α* radiation (40 kV, 40 mA) and a graphite monochromator in a reflection mode (2*θ* = 10–90°, step = 0.016°, scan speed = 5 s per step). Structural refinement of the samples was carried out by using Rietica software. Magnetic susceptibility measurements were carried out using a Quantum Design MPMS5 magnetometer in the temperature range 5–300 K. Approximately 20–40 mg of material was weighed accurately into a gelatin. Measurements were made on warming in a field of 1 kOe, first after cooling in zero field (ZFC) and then again after cooling in the measuring field (FC). The electrical resistivity and thermoelectric property were measured using a Quantum Design physical properties measurement system (PPMS). The electric structure was obtained from first-principles density functional theory (DFT) in the generalized gradient approximation (GGA) according to Perdew *et al*. [23], which were calculated using the plane-wave projector augmented method as implement in the Vienna *ab initio* simulation package (VASP). An energy cut-off of 520 eV was used. The convergence criterion energy was set to be 10^−6^ eV per unit cell and the forces on all relaxed atoms were less than 0.01 eV Å^−1^.

## 3. Results and discussion

**Figure 1.**
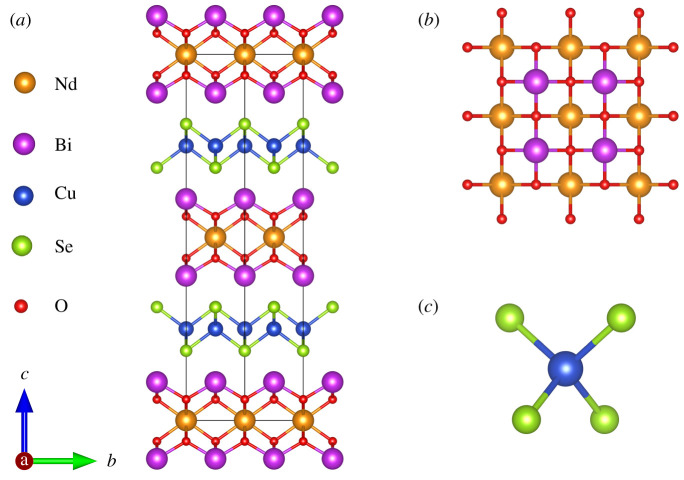
(*a*) The crystal structure of Bi_2_NdO_4_Cu_2_Se_2_; (*b*) the Bi_2_LnO_4_ layers from a vertical view; (*c*) the Cu_2_Se_2_ tetrahedral structure.

The structure of Bi_2_LnO_4_Cu_2_Se_2_ is shown in figure 1, which can be described as stacking of edge-shared Cu_2_Se_2_ tetrahedron layers with Bi_2_LnO_4_ layers alternatively along the c-axis. The Bi_2_LnO_4_ layer can be described as ‘[M_3_O_4_]^+^’ layer, which is a double fluorite-type slab. The [M_3_O_4_] units have been observed in several series of copper oxide superconductors, for example (Ce,Ln)_3_Sr_2_Cu_3_O_11_ and (Y,Ce)_3_SrCuFeO_9_ [24–26]. On the other hand, this type of oxyselenides has a similar structure to the oxyhalides. The structure of BiOCuSe is derived from that of PbFCl or BiOCl through the replacement of chloride by selenide and the incorporation of the Cu^+^ ions into tetrahedral sites coordinated by selenide ions. Thus, the ZrSiCuAs structure of La(Bi)OCuS(Se) is also often described as the stuffed or filled PbFCl structure. The relation between Bi_2_NdO_4_Cl and Bi_2_NdO_4_Cu_2_Se_2_ is different from that of BiOCl and BiOCuSe (electronic supplementary material, figure S1). In fact, the unit cell of Bi_2_NdO_4_Cu_2_Se_2_ can be regarded as double units of BiOCuSe, which are connected by the vertical Bi-site atoms but placed in opposite directions along c-axis. Figure 1*b* shows the Bi_2_LnO_4_ layers from a vertical view. The Ln ions located in square-planar environments in this compound, which provide a model of two-dimensional physical properties of the rear earth element. Figure 1*c* shows the Cu_2_Se_2_ tetrahedral structure.

**Figure 2.**
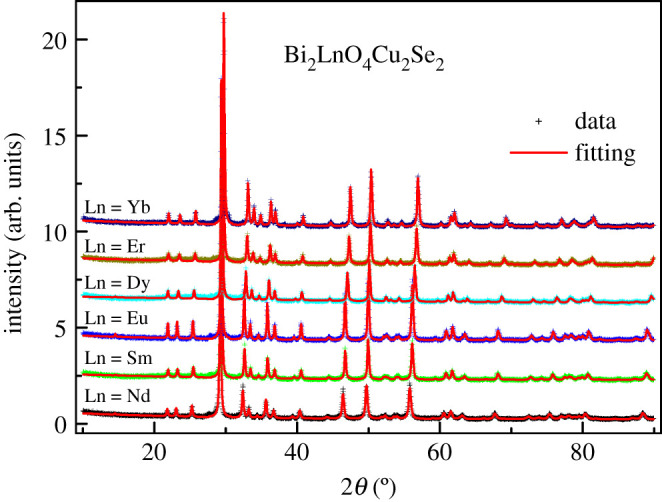
The XRD patterns of Bi_2_LnO_4_Cu_2_Se_2_.

We successfully synthesized a series of Bi_2_LnO_4_Cu_2_Se_2_ (Ln = Nd, Sm, Eu, Dy, Er, Yb), in which the Nd, Sm analogues were first synthesized by Evans *et al*. [21] and the Eu, Dy, Er, Yb analogues are reported for the first time. All the samples are black, which is consistent with their electric transport properties in the following. Figure 2 shows the powder X-ray diffraction (XRD) patterns of Bi_2_LnO_4_Cu_2_Se_2_, scanning over a 2*θ* range of 10–90° at room temperature. Using the tetragonal structure with the space group of I4/mmm, the XRD patterns for the whole compounds can be fitted very well, which are shown with red lines in figure 2. (The details are shown in the electronic supplementary material.) With the Ln ion changing from Nd to Yb, the diffraction peaks gradually shift to higher angle degree, suggesting a contraction of the lattice. The results obtained from the refinement of the X-ray diffraction data are shown in tables 1–3. It can be seen that when the Ln changes from Nd to Yb, the lattice parameters a and c decrease correspondingly, which can be explained by the decrease of the ionic sizes of Ln. Bond lengths provide information about the nature of the chemical bonds. The measured bond length (d) of an ideal ionic bond is close to the estimated bond length given by the sum of the ionic radii of a cation (d_c_) and a neighbouring anion (d_a_). The length of the Cu-Se bond was nearly equal in Bi_2_LnO_4_Cu_2_Se_2_ ranging from 2.42 to 2.45 Å. But the length of Cu-Se bond is shorter than that in BiOCuSe (2.51 Å) and LaOCuSe (2.52 Å) [27]. The Cu ion in the semiconducting BiOCuSe and LaOCuSe is monovalent. The Cu in Bi_2_LnO_4_Cu_2_Se_2_ should be in a higher valence state than in BiOCuSe, because the ionic radii of Cu^2+^ is shorter than that of Cu^+^. The bond length indicates that the Cu cation provides more electrons to the neighbouring Se anion. Our previous results have indicated the existence of a mixed-valence state of Cu^2+^/Cu^+^ in Bi_2_YO_4_Cu_2_Se_2_, which causes the metallic behaviour in this compound. The Bi-O bond lengths in Bi_2_LnO_4_Cu_2_Se_2_ are shorter than that in BiOCuSe (2.33 Å) [27], which suggests the Bi-O has greater covalent character. On the contrary, the Bi-Se bond in Bi_2_LnO_4_Cu_2_Se_2_ lengths are longer than that in BiOCuSe (3.23 Å) [27], indicating the Bi-Se bond are weaker. These observations indicate the two-dimensional nature of Bi_2_LnO_4_Cu_2_Se_2_ is stronger than that in BiOCuSe.

**Table 1.** Fractional atomic coordinates of Bi_2_LnO_4_Cu_2_Se_2_.

atom	*x*	*y*	*z*
Bi	0.5	0.5	z(Bi)
Ln	0.5	0.5	0.5
O	0	0.5	z(O)
Cu	0	0.5	0.25
space group	I4/mmm

**Table 2.** Results of powder X-ray diffraction and Rietveld refinements for Bi_2_LnO_4_Cu_2_Se_2_ at room temperature.

formula	Bi_2_NdO_4_Cu_2_Se_2_	Bi_2_SmO_4_Cu_2_Se_2_	Bi_2_EuO_4_Cu2Se_2_	Bi_2_DyO_4_Cu_2_Se_2_	Bi_2_ErO_4_Cu_2_Se_2_	Bi_2_YbO_4_Cu_2_Se_2_
molecular weight	911.210	917.330	918.934	929.470	934.230	940.010
lattice	tetragonal	tetragonal	tetragonal	tetragonal	tetragonal	tetragonal
space group	I4/mmm	I4/mmm	I4/mmm	I4/mmm	I4/mmm	I4/mmm
a (Å)	3.9175(3)	3.9003(2)	3.8957(2)	3.8695(2)	3.8570(3)	3.844(2)
c (Å)	24.525(2)	24.495(2)	24.439(1)	24.435(2)	24.440(2)	24.424(2)
V (Å^3^)	376.38(5)	372.62(4)	370.35(3)	365.87(3)	363.58(4)	360.89(4)
D (g cm^−3^)	8.037	8.172	8.237	8.433	8.530	8.647
Rp (%)	7.837	7.058	5.390	7.534	8.004	7.666
Rwp (%)	7.632	5.957	4.746	5.911	6.518	6.331
*χ*2	2.437	1.592	1.840	1.760	1.940	2.421
$R_{p}=\sum |y_{io}-y_{ic}|/\sum |y_{io}|$, $R_{wp}={\left[\sum w_{i}{\left(y_{io}-y_{ic}\right)}^{2}/\sum w_{i}y_{io}^{2}\right]}^{1/2}$, $\chi^{2}=\sum [w_{i}{\left(y_{io}-y_{ic}\right)}^{2}/(N-P_{1}-P_{2})]$

**Table 3.** Fractional atomic coordinates, bond length and bond angles determined by the refinement of X-ray diffraction data.

formula	Bi_2_NdO_4_Cu_2_Se_2_	Bi_2_SmO_4_Cu_2_Se_2_	Bi_2_EuO_4_Cu_2_Se_2_	Bi_2_DyO_4_Cu_2_Se_2_	Bi_2_ErO_4_Cu_2_Se_2_	Bi_2_YbO_4_Cu_2_Se_2_
Z(Bi)	0.8951(1)	0.8960(1)	0.8958(1)	0.8973(2)	0.8980(2)	0.8987(1)
Z(Se)	0.3097(3)	0.3099(3)	0.3111(2)	0.3104(3)	0.3100(3)	0.3116(2)
Z(O)	0.9427(9)	0.9456(9)	0.9442(6)	0.947(1)	0.948(1)	0.9476(9)
d_(Bi-O)_(Å)	2.28(1)	2.30(1)	2.277(8)	2.28(2)	2.29(2)	2.27(1)
d_(Bi-Se)_(Å)	3.472(4)	3.472(4)	3.445(3)	3.462(5)	3.474(5)	3.453(4)
d_(Ln-O)_(Å)	2.41(1)	2.36(1)	2.377(9)	2.30(2)	2.30(2)	2.30(1)
d_(Cu-Se)_(Å)	2.445(4)	2.440(4)	2.453(3)	2.434(5)	2.422(5)	2.440(3)
Angle_(O-Bi-O)_ (degree)	118.5(9) × 2	116(1) × 2	117.5(7) × 4	116(1) × 2	115(2) × 2	116(1) × 2
	74.8(4) × 4	73.8(5) × 4	74.4(3) × 4	73.7(6) × 4	73.1(6) × 4	73.6(5) × 4
Angle_(Se-Cu-Se)_ (degree)	106.5(3) × 2	106.1(3) × 2	105.1(2) × 2	105.3(3) × 2	105.5(3) × 2	104.0(2) × 2
	111.0×4	111.2(1) × 4	111.7(1) × 4	111.6(2) × 4	111.5(2) × 4	112.3(1) × 4

**Figure 3.**
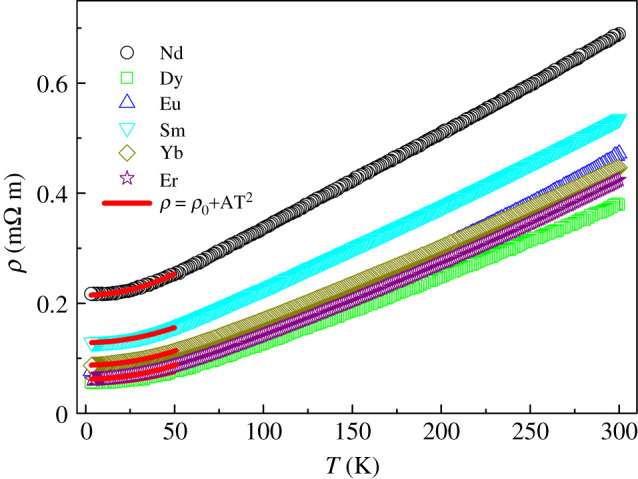
The temperature dependence of the resistivity of Bi_2_LnO_4_Cu_2_Se_2_ from 2 to 300 K. The red lines are the Fermi-liquid fitting of the resistivity at low temperature.

Recently, we studied the physical properties of the analogue Bi_2_YO_4_Cu_2_Se_2_. The theoretical calculation indicated that the ground state of this compound is the quasi-two-dimensional metal state. The states of the valence band maximum are mainly composed of antibonding Cu-3d/Se-4p states and the conduction band are mainly Bi-6p/O-2p states. When the transferred valence electrons from the blocking layer to Cu_2_Se_2_ layer are less than two per layer, the compound would exhibit metallic behaviours. The electric transport properties of Bi_2_LnO_4_Cu_2_Se_2_ are shown in figure 3. All these compounds show metallic behaviours in the measured temperature region, which agrees with the conclusion we obtained from theoretical calculations. For all the samples, the resistance drops linearly from 300 to 100 K. The influence of Ln atoms to the transport properties is mainly embodied in the influence of the structure of the Cu-Se layer. As the Ln atom changed from Nd to Yb, the size of which is decreasing, the angle of Se-Cu-Se and the distance of Cu-Cu tend to decrease. As a result, the electrons transferred easily in Cu-Se layers. This can be demonstrated by the decreasing resistivity and smaller temperature coefficient of resistivity as Ln changing from Nd to Yb. At low-temperature region, the resistance is satisfied with the Fermi-liquid behaviour in the ground state and we fitted the resistance curve using the equation *ρ* = *ρ*_0_ + AT^2^, where *ρ*_0_ is the residual resistivity, and the coefficient A represents the inelastic scattering between electrons and is generally proportional to the square of the effective electron mass. (The values of the fitted *ρ*_0_ and A are shown in electronic supplementary material, table S1.) We also measured the thermoelectric transport properties of all the samples. The Seebeck coefficients are positive in the whole measured temperature region, indicating the major carriers are holes for all the samples. (The measured Seebeck coefficient S, thermal conductivity *κ*, electrical conductivity *σ* and figure of merit ZT (ZT = S^2^T*σ*/*κ*) at room temperature are shown in electronic supplementary material, table S2.)

We measured the magnetic properties of the samples. Plots of the temperature dependence of the magnetic susceptibility for Bi_2_LnO_4_Cu_2_Se_2_ (Ln = Sm, Nd, Eu, Dy, Er, Yb) are shown in electronic supplementary material, figure S3. These samples have relatively high susceptibility compared with Bi_2_YO_4_Cu_2_Se_2_. The order of susceptibility from large to small is Dy, Er, Yb, Nd and Sm, which is related to the occupation of the 4f orbital in the Ln ions. All the measured samples exhibit paramagnetic behaviour in the measured temperature region from 5 to 300 K. For Dy and Eu analogues, the inverse susceptibility curves are relatively linear with temperatures nearly in the whole measured temperature region, which could be fitted by the normal Curie–Weiss law, *χ* = C/(T − *θ*_p_). For Nd and Yb analogues, their susceptibility curves obey the Curie–Weiss law only in the higher temperature regions, and the inverse susceptibility curves deviate from linearity at low temperatures. The magnetic behaviour of Bi_2_SmO_4_Cu_2_Se_2_ is not remarkable and the inverse susceptibility curve for Bi_2_SmO_4_Cu_2_Se_2_ is convex, indicating the itinerant electron susceptibility is non-negligible. We fitted the susceptibility curve in the whole measured temperature range using the modified Curie–Weiss law, *χ* = C/(T − *θ*_p_) + *χ*_0_. We calculated the effective moment from the fitted parameters which are plotted in electronic supplementary material, figure S3. These values are all consistent with the expected values for the trivalent Ln free ions, which can be expressed as $\mu =\text{g}\sqrt{J(J+1)}$. All the Weiss constants are negative, suggesting antiferromagnetic exchange interactions, but further measurements are required for more detailed interpretation.

**Figure 4.**
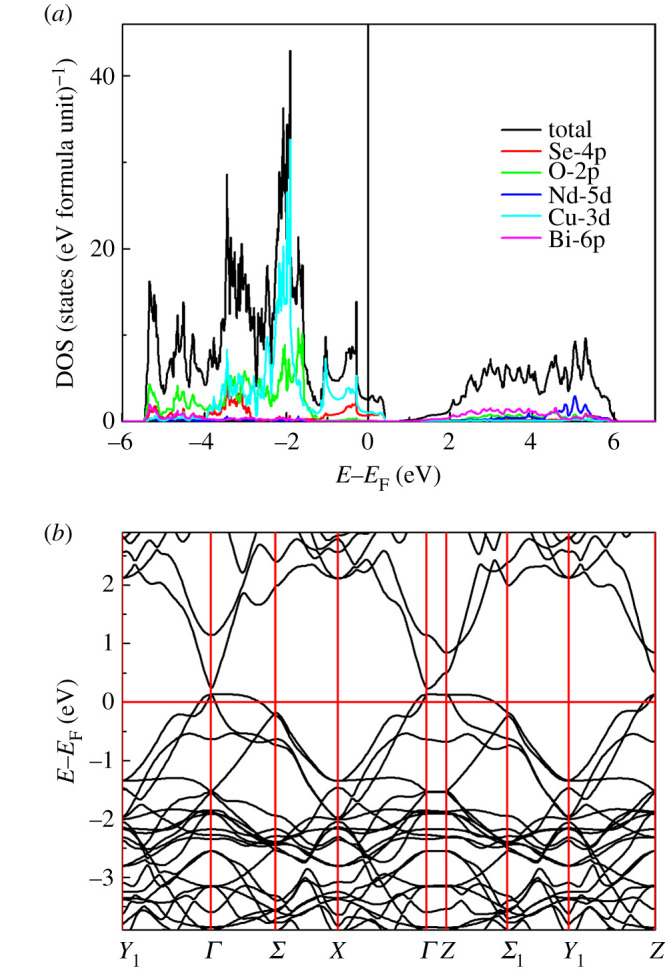
(*a*) The DOS and (*b*) the band structure of Bi_2_NdO_4_Cu_2_Se_2_.

In order to verify the metallic ground state, we studied the density of states (DOS) and the band structure of Bi_2_NdO_4_Cu_2_Se_2_ calculated using DFT. The crystal structures of Bi_2_NdO_4_Cu_2_Se_2_ were optimized with respect to the lattice parameters and atomic positions. All the optimized lattice parameters and atomic coordinates are in good agreement with the experimental observation. The calculated DOS of Bi_2_NdO_4_Cu_2_Se_2_ is shown in figure 4*a*. There is finite DOS at the Fermi level, indicating the metallic ground state. The valence band maximum of Bi_2_NdO_4_Cu_2_Se_2_ mainly consists of Cu-3d and Se-4p electrons. The conduction band minimum is mainly built up of Bi-6p and O-2p electrons. Figure 4*b* shows the band structure of Bi_2_NdO_4_Cu_2_Se_2_. There are two bands crossing the *E*_F_ and the *E*_F_ locates at the valence band, indicating the metallic ground state and the hole-type carriers. There is a small gap between the valence band and conduction band at *Γ* point. Other Bi_2_LnO_4_Cu_2_Se_2_ have similar band structures which are not shown here.

## 4. Conclusion

In summary, we have synthesized a new series of layered oxyselenides Bi_2_LnO_4_Cu_2_Se_2_ through solid-state reaction. Their crystalline structures were studied through X-ray diffraction experiment. These compounds were crystalline in tetragonal structure with space group I4/mmm. All these compounds exhibit metallic transport property with hole-type carriers, which is verified by the DFT calculation. All the samples exhibit paramagnetic behaviour and no magnetic transition was found from 5 to 300 K. The new Bi_2_LnO_4_-type block in these compounds may give people some enlightenment in synthesizing new iron-based superconductors or other layered compounds.

## Supplementary Material

The crystal structure, XRD fitting and room temperature thermoelectric properties of Bi2Ln2O4Cu2Se2

Reviewer comments

## Data accessibility

The datasets supporting this article have been uploaded as part of the electronic supplementary material.
