# Supplementary material for: Synthesis, structure and physical properties of the new layered oxyselenides Bi2LnO4Cu2Se2 (Ln = rare earth)
Source: R Soc Open Sci. 2020 Oct 28;7(10):201078. doi: 10.1098/rsos.201078 (PMC7657882; doi:10.1098/rsos.201078)
Supplement: The crystal structure, XRD fitting and room temperature thermoelectric properties of Bi2Ln2O4Cu2Se2 [file rsos201078supp1.docx]

**ELECTRIC SUPPLEMENT INFORMATION**

**Synthesize, structure and physical properties of the new layered oxyselenides Bi_2_LnO_4_Cu_2_Se_2_ (Ln = rare earth)**

Shugang Tan*^a^, Chenhao Gao^a^, , Cao Wang^a^, Qiang Jing^a^, Tong Zhou^a^, Guangchao Yin^a^, Meiling Sun^a^, Fei Xing^a^, Rui Cao^b^, and Yuping Sun*^a^

^a^School of Physics and Optoelectronic Engineering, Shandong University of Technology, Zibo 255000, People’s Republic of China

^b^Office of International Cooperation and Exchange, Shandong University of Technology, Zibo 255000, People’s Republic of China


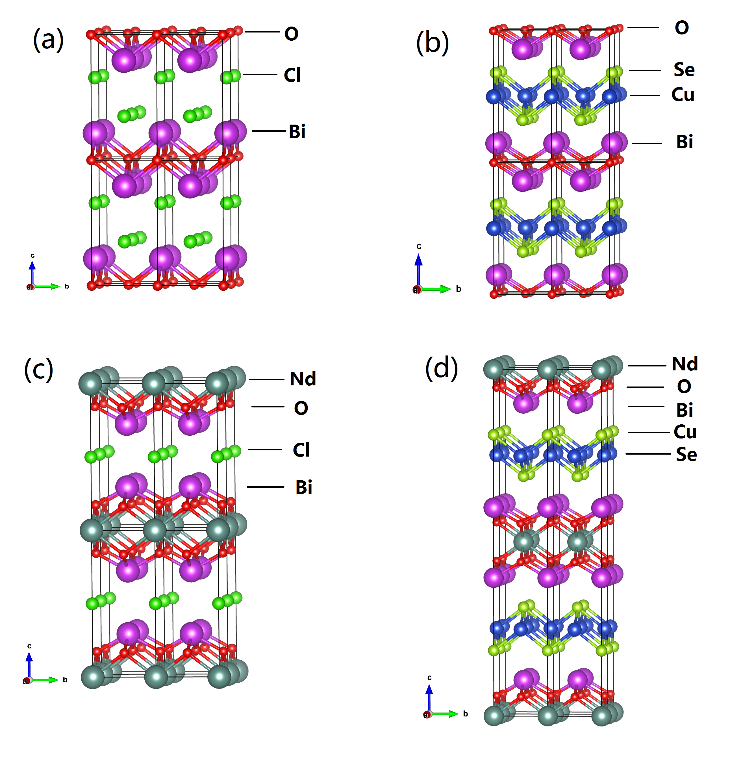


Figure S1: The crystal structure of (a)BiOCl, (b)BiOCuSe,(c)Bi_2_NdO_4_Cl, and (d)Bi_2_NdO_4_Cu_2_Se_2_


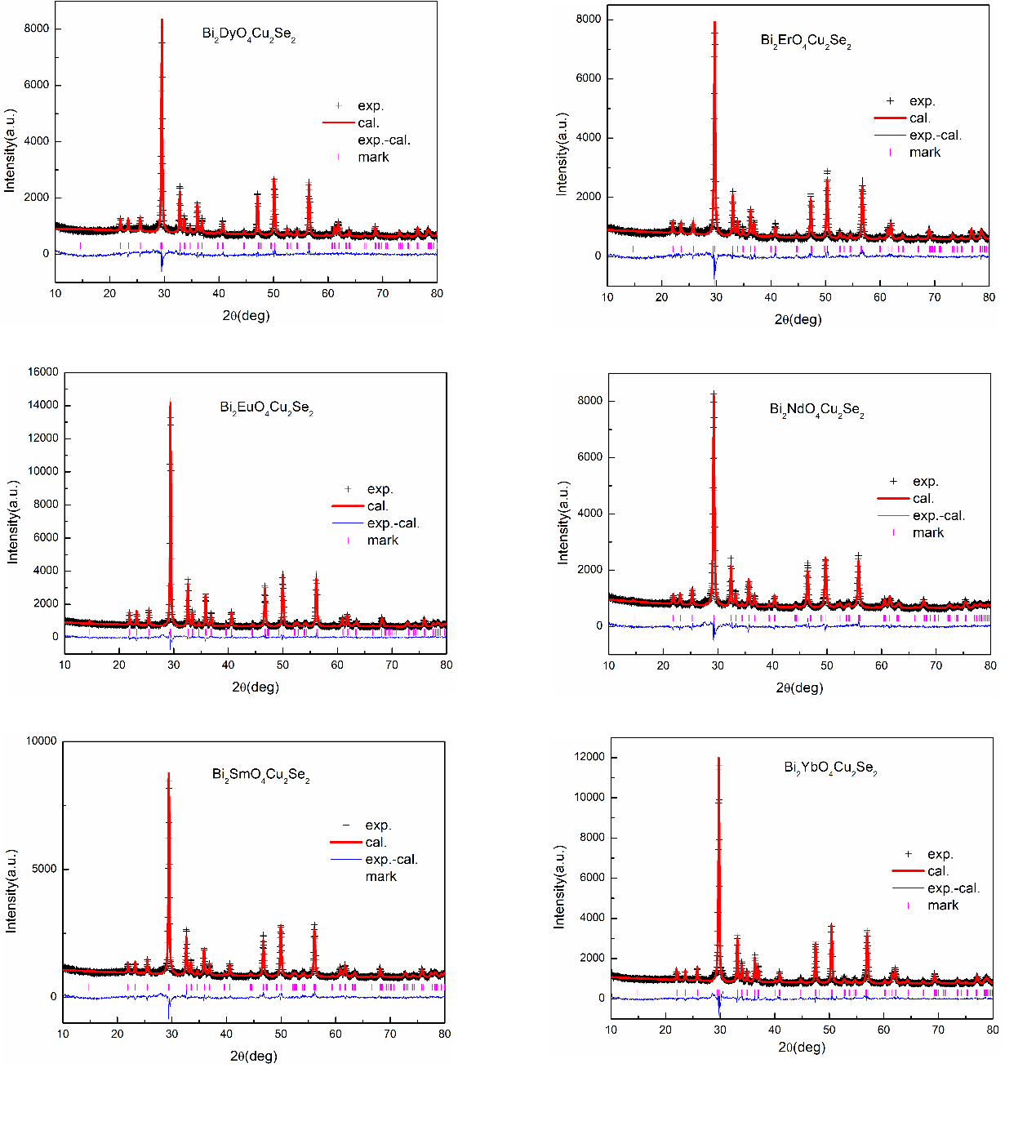


Figure S2: The refinement of the XRD data of Bi_2_LnO_4_Cu_2_Se_2_.

Table S1: The values of the thermoelectric parameters at room temperature.

| Sample | S (μV/K) | κ (W/K m) | σ (10^3^S/cm) | ZT |
| --- | --- | --- | --- | --- |
| Bi_2_NdO_4_Cu_2_Se_2_ | 18.1 | 3.39 | 2.3 | 0.0056 |
| Bi_2_SmO_4_Cu_2_Se_2_ | 16.6 | 3.59 | 1.8 | 0.0042 |
| Bi_2_ErO_4_Cu_2_Se_2_ | 17.4 | 4.00 | 2.7 | 0.0062 |

Table S2: The fitted parameters of the resistivity at low temperature through Fermi liquid equation ρ=ρ_0_+AT^2^.

| formula | Bi_2_NdO_4_Cu_2_Se_2_ | Bi_2_SmO_4_Cu_2_Se_2_ | Bi_2_EuO_4_Cu_2_Se_2_ | Bi_2_DyO_4_Cu_2_Se_2_ | Bi_2_ErO_4_Cu_2_Se_2_ | Bi_2_YbO_4_Cu_2_Se_2_ |
| --- | --- | --- | --- | --- | --- | --- |
| ρ_0_(Ωm) | 2.1×10^-4^ | 1.2×10^-4^ | 7.5×10^-5^ | 5.4×10^-5^ | 6.1×10^-5^ | 8.7×10^-5^ |
| A(Ωm/T^2^) | 1.5×10^-8^ | 1.1×10^-8^ | 1.0×10^-8^ | 8.5×10^-9^ | 1.0×10^-8^ | 1.0×10^-8^ |





Figure S3: The temperature dependence of the magnetic susceptibility for Bi_2_LnO_4_Cu_2_Se_2_. The red lines are the fitting curves. The inset in the lower right corner shows the calculated and experimental effective magnetic moment of Ln ions.
